# CT grading combined with inflammatory markers for predicting the efficacy of conservative treatment in acute appendicitis

**DOI:** 10.1016/j.clinsp.2026.100929

**Published:** 2026-04-15

**Authors:** Zhizun Lin

**Affiliations:** Department of Emergency Surgery, The Second Affiliated Hospital of Fujian Medical University, Quanzhou, Fujian, China

**Keywords:** Acute appendicitis, CT imaging grading, Inflammatory markers, Prognosis

## Abstract

**Aims:**

To investigate the clinical characteristics of patients with first-episode acute appendicitis and explore the value of CT imaging grading combined with inflammatory markers in evaluating the cure and relapse of conservative treatment.

**Methods:**

A retrospective cohort study was conducted to collect the clinical data of 482 patients with acute appendicitis diagnosed in the Emergency Department of the Second Affiliated Hospital of Fujian Medical University from October 2021 to February 2024. The imaging features of these patients were evaluated using Computed Tomography (CT) scans. Descriptive analysis, chi-square test, ordinal logistic regression model, Kaplan-Meier curve, and COX regression analysis were used to analyze the data.

**Results:**

Elevated levels of Procalcitonin (PCT) and C-Reactive Protein (CRP) were associated with higher CT grades. Acute appendicitis patients with higher CT grades had a poor prognosis with antibiotic treatment. CT grade was a predictor of the prognosis of antibiotic treatment, and a high CT grade was an independent risk factor for relapse.

**Conclusion:**

CT grade combined with inflammatory markers (PCT and CRP) can effectively predict the prognosis of conservative treatment for acute appendicitis, and CT grade can be used to predict the risk of relapse.

## Highlights

• CT imaging grading combined with PCT/CRP effectively predicts conservative treatment prognosis for acute appendicitis.
• Higher CT grades correlate with elevated PCT/CRP and poorer antibiotic therapy outcomes in acute appendicitis patients.
• CT grade is an independent risk factor for relapse, with higher grades shortening relapse-free survival time.
• NLR, white blood cell/neutrophil counts also link to increased conservative treatment failure risk in acute appendicitis.
• Low CT grades suit conservative treatment; high grades warrant prioritized surgical intervention for acute appendicitis.

## Introduction

Acute appendicitis is one of the most common causes of acute abdominal pain, with an incidence in developed countries ranging from 5.7 to 50 cases per 100,000 inhabitants per year.^1^^,^^2^ It affects all age groups, and treatment options include conservative therapy and surgical intervention. Traditionally, surgical treatment has been the first choice for acute appendicitis, but patients with first-episode acute appendicitis often refuse surgery for various reasons and prefer antibiotic therapy. For these patients, clinical indicators that can clearly predict the prognosis of conservative treatment are crucial for clinical decision-making.

Two large-scale randomized controlled trials^3^^,^^4^ have validated the effectiveness of antibiotic therapy for CT-diagnosed simple appendicitis. CT scanning has become an important tool for the diagnosis of acute appendicitis, and multi-slice spiral CT technology has significantly improved the diagnostic accuracy. Previous studies have shown that CT has higher specificity and sensitivity in the diagnosis of acute appendicitis compared with ultrasound.^5^, ^6^, ^7^ Multi-slice spiral CT can not only accurately diagnose acute appendicitis but also classify it according to the severity, providing a basis for clinical treatment.

However, few studies have explored the relationship between CT imaging grading and the efficacy of conservative treatment or post-cure relapse in acute appendicitis. This study aims to investigate the value of CT imaging grading combined with inflammatory parameters in predicting the prognosis and relapse of conservative treatment for acute appendicitis.

## Materials and methods

### Ethics

The Institutional Research Ethics Committee of the Second Clinical Medical College of Fujian Medical University has approved this study (Ethics Approval No: Ethical Rev. of 2nd Affil. Hosp. of Fujian Med. Univ., n°052, 2025). All patients have provided written informed consent for the use of their clinical data (including demographic data, CT imaging findings, inflammatory marker test results, and follow-up records) in this study. During the study, all patients' clinical data have been de-identified (e.g., removing personal identifiers such as names, hospital numbers, and contact information), and relevant privacy protection regulations have been strictly followed to ensure that the rights and interests of participants are not violated.

This Study follows the STROBE Guidelines.

### General data

A total of 482 patients with acute appendicitis admitted to the hospital from October 2021 to February 2024 were enrolled, including 275 males and 207 females, aged 18- to 85-years (mean age: 42.26±14.33 years). This study was approved by the Ethics Committee of the hospital. All patients underwent 64-slice spiral CT scanning within 12-h of admission and were requested conservative treatment after the examination.

### Inclusion and exclusion criteria

Inclusion criteria: 1) Clinical symptoms such as nausea, vomiting, and tenderness at the McCartney's point; 2) Meeting the indications for CT examination; 3) All patients provided informed consent for the use of their clinical data for research purposes.

Exclusion criteria: 1) Comorbid malignant tumors; 2) Pregnant or lactating women; 3) Comorbid severe organ dysfunction; 4) Comorbid gastrointestinal bleeding.

## Methods

### Treatment protocol

Medical records were collected to observe the effect of conservative treatment. The treatment included third-generation cephalosporins, with or without nitroimidazole combination antibiotic therapy, combined with fasting or liquid diet, intravenous infusion of energy (glucose or glucose saline), electrolytes, and symptomatic treatment. The surgical team conducted at least 2 repeated physical examinations within 24‒48 h after admission, and rechecked the Complete Blood Count (CBC) and inflammatory markers at 24-h and 48-h after admission, respectively. Experienced general surgery surgeons evaluated the therapeutic effect during the therapy. If necessary, a re-examination with CT was conducted, and conservative treatment was terminated in a timely manner to switch to surgical treatment.

### Examination method

A Philips 64-slice spiral CT scanner was used for scanning. Patients were in the supine position without a contrast agent, and breath-holding was required during scanning. The scanning range was from the third lumbar vertebra to the pubic symphysis. Multi-planar reconstruction and maximum intensity projection were performed after scanning to observe the lesions comprehensively.

C-Reactive Protein (CRP): Detected by the immunoturbidimetric method, using a Roche automatic biochemical analyzer, with the supporting reagent being the Roche CRP test kit.

Procalcitonin (PCT): Detected by the fluorescent immunochromatographic method, using a Roche Cobas e602 electrochemiluminescence immunoanalyzer, with the reagent being the Roche Diagnostics PCT test kit.

Blood Cell Count Test: Detected by the combination of the electrical impedance method and laser scattering method, using a Mindray automatic hematology analyzer.

### Observation indicators

CT imaging grading standards

See Table 1 for details.

**Table 1:** CT imaging grading of appendicitis.

Grade	Appendicitis
Ⅰ	The appendix is filled with fluid, with a diameter of 6.0‒7.9 mm, and the periappendiceal fat space is clear, suggesting possible appendicitis.
Ⅱ	The appendix is filled with fluid, with a diameter > 6.0 mm, increased wall thickness, but no periappendiceal exudation, indicating simple appendicitis.
Ⅲ	The appendix is filled with fluid, with a diameter > 6.0 mm, increased wall thickness, and periappendiceal exudation, indicating appendicitis with periappendicitis; if the appendix is not visualized, but fecaliths appear in the appendiceal area with cecum peri‑inflammation, it is also classified into this grade.
Ⅳ	The appendix is filled with fluid, with a diameter > 6.0 mm, increased wall thickness, unclear periappendiceal boundary, and periappendiceal effusion, indicating hemorrhagic or gangrenous appendicitis.
Ⅴ	Grade 5: Periappendiceal abscess or inflammatory mass, indicating appendiceal abscess.

Treatment outcomes

Cure: Disappearance of abdominal pain, no local tenderness, and normalization of white blood cell count and neutrophil percentage.

Failure: Worsening abdominal pain, obvious tenderness and rebound tenderness, systemic inflammatory response, and persistent elevation of white blood cells and neutrophils.

Relapse: Re-diagnosis of appendicitis during the follow-up period after successful conservative treatment of the first episode.

### Follow-up

The last follow-up was in April 2025, and the final treatment and relapse status of all patients were known. The follow-up period was defined as the time from the end of the first episode treatment to the date of relapse or the last follow-up.

### Statistical methods

SPSS 24.0 software was used for data analysis. Descriptive analysis and chi-square test were used to analyze the correlation between treatment outcomes and clinical parameters. An ordinal logistic regression model was adopted to explore the relationship between CT grade and multiple independent variables. The Kaplan-Meier curve was used for survival comparison. COX regression analysis was performed for Univariate and Multivariate Analysis to control confounding factors. A *p* < 0.05 was considered statistically significant. Receiver Operating Characteristic (ROC) analysis was used to determine the cut-off value of parameters for group differentiation, and ROC curves were drawn based on sensitivity and specificity at each value.

## Results

### Clinical characteristics of patients with acute appendicitis

CT grade distribution

**Fig. 1:**
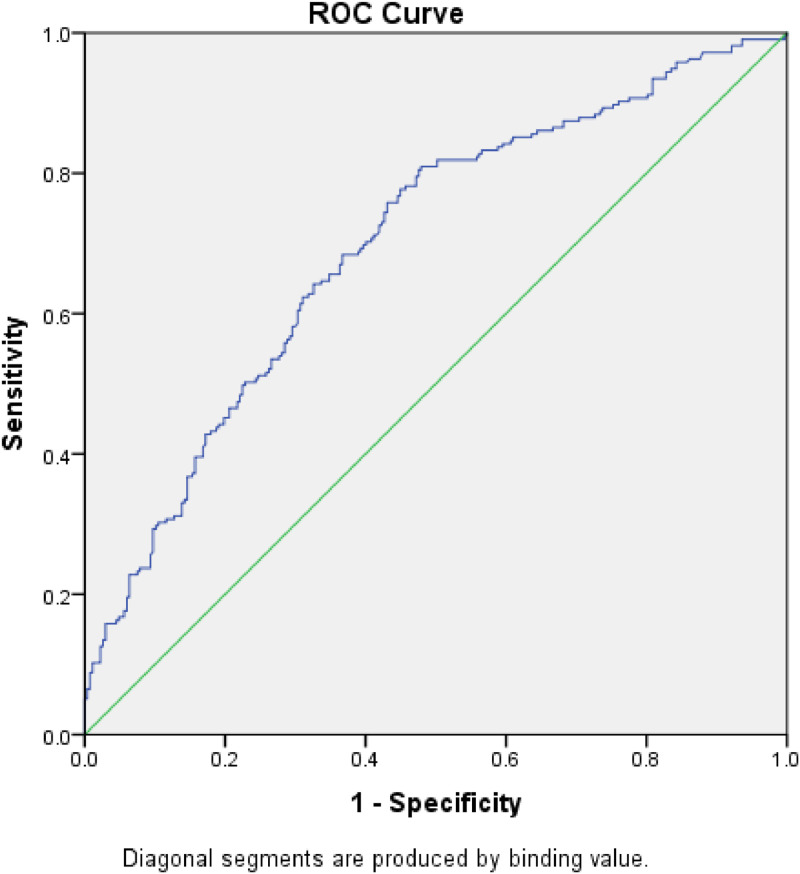
ROC curve for the success and failure of conservative treatment of acute appendicitis. The area under ROC curve: 0.698. 95 % CI 0.651‒0.745; p-value < 0.001.

The CT grades of 482 acute appendicitis patients mainly concentrated in Grade 2 (39.0 %, 188/482) and Grade 3 (34.2 %, 165/482) (Table 2). The recommended cut-off value of Neutrophil-to-Lymphocyte Ratio (NLR) for conservative treatment of acute appendicitis was determined by ROC curve analysis. The recommended cut-off value of NLR was defined as 5.72 based on the point with the most significant sensitivity (0.809) and specificity (0.521) on the ROC curve (Fig. 1). The area under the ROC curve was 0.698 (95 % CI 0.651‒0.745; *p* < 0.001).

**Table 2:** The composition of patients with different CT grades.

Grading of CT images	N/Total	The composition ratio among groups
1	66/482	13.7 %
2	188/482	39.0 %
3	165/482	34.2 %
4	50/482	10.4 %
5	13/482	2.7 %

Ordinal logistic analysis of CT grade

CRP and PCT levels had a significant impact on CT grading (*p* < 0.001). The estimated value of the PCT level was −0.844, indicating that the lower the PCT level, the higher the possibility of lower CT grading. Similarly, CRP level also had a significant impact on CT grading (*p* < 0.001), with an estimated value of −0.955, meaning that the lower the CRP level, the higher the possibility of lower CT grading. Variables such as age, gender, and white blood cell count had no significant effect on CT grading (*p* > 0.05), indicating that these factors could not effectively predict changes in CT grading in this study (Table 3).

**Table 3:** Logistic analysis of clinical features and CT grading.

Parameter		Estimated value	OR (95 % CI)	p
Threshold	CT1	−4.185	0.015 (0.003‒0.092)	<0.001
	CT2	−1.933	0.145 (0.025‒0.849)	0.032
	CT3	0.115	1.122 (0.192‒6.551)	0.898
	CT4	1.898	6.675 (1.075‒41.437)	0.042
Gender				
Male			1 (Referent)	
Female		−0.238	0.788 (0.561‒1.108)	0.170
Age (year)				
≥ 60			1 (Referent)	
< 60		−0.273	0.761 (0.442‒1.312)	0.326
NLR				
≥ 5.72			1 (Referent)	
< 5.72		−0.489	0.613 (0.407‒0.924)	0.019
Leukocyte count (10^9/L)				
≥ 10			1 (Referent)	
< 10		−0.342	0.711 (0.390‒1.296)	0.265
Neutrophil count (10^9/L)				
≥ 6.3			1 (Referent)	
< 6.3		−0.233	0.792 (0.380‒1.650)	0.534
Lymphocyte count (10^9/L)				
< 0.8		−0.369	0.691 (0.118‒4.036)	0.682
< 4 且 ≥ 0.8		−0.590	0.554 (0.103‒2.997)	0.493
≥4			1 (Referent)	
PCT				
≥ 0.05			1 (Referent)	
< 0.05		−0.844	0.430 (0.295‒0.626)	<0.001
CRP				
≥ 6			1 (Referent)	
< 6		−0.955	0.385 (0.265‒0.559)	<0.001

Dependent variable: CT grading.

Model: [ %1], CT grading.

This parameter is set to zero because it is redundant.

Fixed at the displayed value.

### Correlation between clinical characteristics and conservative treatment outcomes

Clinical indicators such as NLR, white blood cell count, neutrophil count, PCT, CRP, and CT grading were significantly correlated with conservative treatment outcomes. Higher levels of NLR, PCT, CRP, white blood cell count, neutrophil count, and CT grade were associated with a higher risk of conservative treatment failure. Gender, lymphocyte count, and age were not related to treatment outcomes (Table 4).

**Table 4:** Characteristics of patients with appendicitis according to treatment outcomes.

Clinicopathologic characteristics	Treatment Outcomes			
	Succeed	Failure	Total	p
Gender				0.520
Male	142 (51.6 %)	133 (48.4 %)	275	
Female	113 (54.6 %)	94 (45.4 %)	207	
Age (year)				0.076
≥ 60	21 (41.2 %)	30 (58.8 %)	51	
< 60	234 (54.3 %)	197 (45.7 %)	431	
NLR				<0.001
≥ 5.72	129 (42.9 %)	172 (57.1 %)	301	
< 5.72	126 (69.6 %)	55 (30.4 %)	181	
Leukocyte count (10^9/L)				0.001
≥ 10	184 (48.8 %)	193 (51.2 %)	377	
< 10	71 (67.6 %)	34 (32.4 %)	105	
Neutrophil count (10^9/L)				<0.001
≥ 6.3	210 (50.4 %)	207 (49.6 %)	417	
< 6.3	48 (73.8 %)	17 (26.2 %)	65	
Lymphocyte count (10^9/L)				0.069
< 0.8	28 (41.2 %)	40 (58.8 %)	68	
< 4且 ≥ 0.8	224 (55.2 %)	182 (44.8 %)	406	
≥ 4	3 (37.5 %)	5 (62.5 %)	8	
PCT				<0.001
≥ 0.05	42 (18.5 %)	185 (81.5 %)	227	
< 0.05	213 (83.5 %)	42 (16.5 %)	255	
CRP				<0.001
≥ 6	86 (31.9 %)	184 (68.1 %)	270	
< 6	169 (79.7 %)	43 (20.3 %)	212	
CT grade				<0.001
1	63 (95.5 %)	3 (4.5 %)	66	
2	118 (62.8 %)	70 (37.2 %)	188	
3	67 (40.6 %)	98 (59.4 %)	165	
4	4 (8 %)	46 (92.0 %)	50	
5	3 (23.1 %)	10 (76.9 %)	13	

### Relapse-free survival (RFS)

**Fig. 2:**
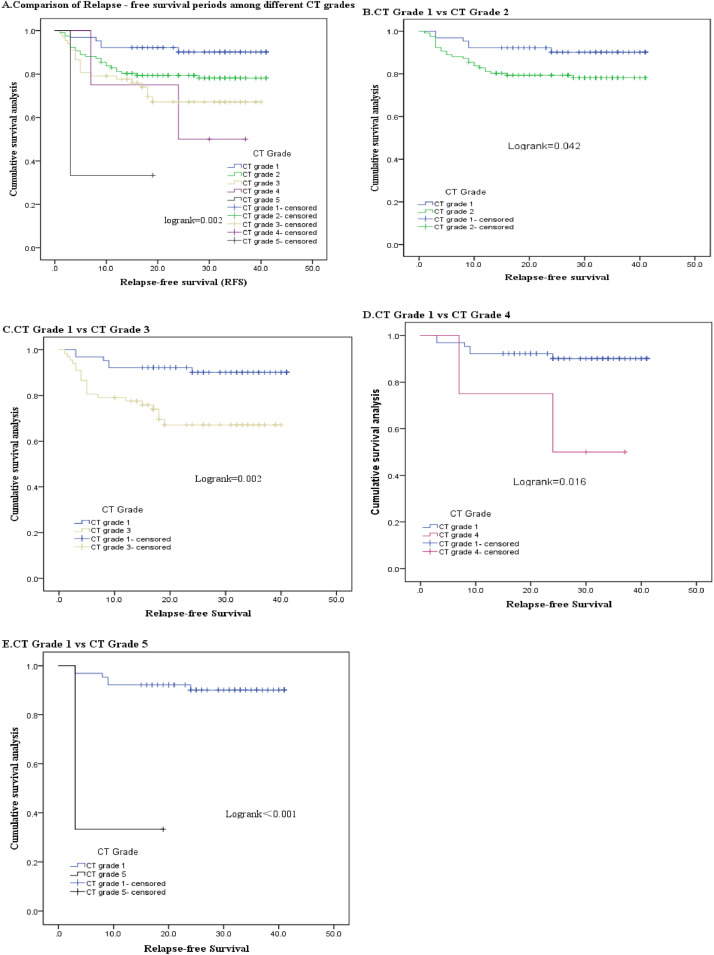
Kaplan-Meier curves of Acute appen*dicitis* according to CT grade. (A) Acute appendicitis patients’ Relapse free survival according to CT grade. (B) The Relapse free survival of CT Grade 1 compared to CT Grade 2. (C) The Relapse free survival of CT grade 1compared to CT Grade 3. (D) The Relapse free survival of CT Grade 1compared to CT Grade 4. (E) The Relapse free survival of CT grade 1compared to CT Grade 5.

Further analysis of relapse in 255 patients with successful conservative treatment showed that the RFS of CT Grade 1 patients was 37.941±1.205 months, CT Grade 2 was 33.733±1.305 months, CT Grade 3 was 29.646±1.941 months, CT Grade 4 was 26.250±6.158 months, and CT Grade 5 was 8.333±4.355 months. It was found that the RFS of acute appendicitis patients became shorter with the increase of CT grade (*p* < 0.05) (Table 5, Fig. 2). The Kaplan-Meier survival curve (Log-rank *p* = 0.002) and COX risk regression model analysis (univariate analysis: HR = 2.515; 95 % CI 1.885‒3.356; *p* < 0.001; multivariate analysis: HR = 1.577; 95 % CI 1.166‒2.133; *p* = 0.003) both indicated that CT grade was an independent prognostic factor for relapse after successful conservative treatment of acute appendicitis, and there were significant differences in RFS among patients with different CT grades. The higher the CT grade, the shorter the RFS and the higher the risk of relapse (Fig. 2, Table 5, Table 6, Table 7). Multivariate analysis emphasized that high CT grade was an independent risk factor for relapse in patients with acute appendicitis after controlling for other risk factors.

**Table 5:** Univariate analysis of clinical variable in relation to RFS in patients with appendicitis.

Clinical Variable	RFS	
	HR (95 % CI)	p
Gender		0.630
Male	0.876 (0.513‒1.498)	
Female	1 (Referent)	
Age (year)		0.506
≥ 60	1 (Referent)	
< 60	1.484 (0.463‒4.752)	
Leukocyte (10^9/L)		0.755
≥ 10	1.099 (0.607‒1.990)	
< 10	1 (Referent)	
Neutrophil (10^9/L)		0.708
≥ 6.3	1.140 (0.574‒2.262)	
< 6.3	1 (Referent)	
Lymphocyte count (10^9/L)		0.045
< 0.8	1 (Referent)	
< 4且 ≥ 0.8	1.215 (0.484‒3.051)	
≥ 4	7.004 (1.355‒36.207)	
PCT		0.137
≥ 0.05	1.603 (0.860‒2.986)	
< 0.05	1 (Referent)	
CRP		0.058
≥ 6	1.671 (0.983‒2.842)	
< 6	1 (Referent)	
NLR		0.995
< 5.72	1 (Referent)	
≥ 5.72	1.002 (0.590‒1.700)	
CT grade		0.006
1	1 (Referent)	
2	2.458 (1.008‒5.993)	0.048
3	3.781 (1.517‒9.425)	0.004
4	5.654 (1.141‒28.020)	0.034
5	13.310 (2.665‒66.474)	0.002

**Table 6:** Multivariate analysis of Clinical variable in relation to RFS in patients with appendicitis.

Clinical Variable	RFS	
	HR (95 % CI)	p
CT grade		
1	1 (Referent)	
2	2.347 (0.955‒5.768)	0.043
3	3.784 (1.517‒9.441)	0.004
4	5.588 (1.127‒27.713)	0.035
5	13.228 (2.647‒66.113)	0.002
Lymphocyte count (10^9/L)		
< 0.8	1 (Referent)	
< 4且 ≥ 0.8	1.226(0.485‒3.099)	0.667
≥ 4	7.155(1.368‒37.431)	0.06

**Table 7:** The survival comparison of different CT grade.

CT Grade	RFS	p
1	37.941±1.205	
2	33.733±1.305	0.042
3	29.646±1.941	0.002
4	26.250±6.158	0.016
5	8.333±4.355	<0.001

## Discussion

Accurate prediction of the prognosis of conservative treatment for acute appendicitis is crucial for formulating reasonable treatment plans in clinical practice. This study provides valuable references for clinicians through the analysis of CT imaging grading combined with inflammatory markers.

From the perspective of inflammatory markers, PCT and CRP play key roles in evaluating the severity of acute appendicitis and the prognosis of conservative treatment. The authors found that the PCT level was a predictor of CT grading in patients with acute appendicitis. This may be because when appendicitis occurs, especially in cases of purulent or gangrenous appendicitis, massive bacterial proliferation and toxin release can exacerbate the systemic inflammatory response, thereby increasing the PCT level in the blood. During the inflammatory process of acute appendicitis, mucosal secretion increases, and the resident bacteria in the appendix begin to multiply rapidly. Impaired blood supply compromises the integrity of the appendix, leading to bacterial invasion and release of bacterial endotoxins,^8^ which in turn induces an elevation of PCT.^9^^,^^10^

Hiromasa Yamashita et al.^11^ found that both PCT and CRP could be used as indicators to predict complications of acute appendicitis. Analysis of clinical data from patients with acute appendicitis showed that PCT and CRP were useful markers for acute appendicitis with abscess or perforation. Persistent elevation or high levels of PCT may indicate that inflammation is not effectively controlled, with risks of appendiceal perforation, abscess formation, and other complications. Lucy Dale^12^ confirmed in her study that PCT levels in patients with Complicated Appendicitis (CAA), such as perforation, gangrene, or necrosis, were significantly higher than those in patients with Uncomplicated Appendicitis (UAA) and various other non-appendiceal lesions, which is consistent with the present finding that higher PCT levels are associated with higher CT grades in acute appendicitis patients.

CRP was also positively correlated with CT grade. Yosuke Sasaki et al.^13^ found that high CRP levels were strong dose-dependent predictors of Complicated Appendicitis (CA). Gurleyik et al.^14^ suggested that CRP < 6 mg/L indicated negative, 6‒35 mg/L indicated non-perforated appendicitis, and > 84 mg/L indicated perforated appendicitis. The present study found that the higher the CRP level, the higher the CT grade and the severity of appendicitis, which is consistent with the results of R Ghimire et al.,^15^ further confirming the value of CRP in evaluating the severity of appendiceal inflammation.

International renowned journals such as The Lancet, JAMA, and Annals of Surgery have successively published multiple prospective multi-center clinical studies confirming the safety and reliability of conservative treatment for uncomplicated appendicitis.^3^, ^4^^,^^16^ CT diagnostic grading has been verified to correlate well with surgical and pathological findings, which play a key role in rapidly predicting the severity and pathological type of acute appendicitis and guiding clinical treatment decisions. Specifically, CT Grades 1–2 correspond to simple appendicitis with inflammation confined to the appendix itself, while Grades 3–5 represent complicated appendicitis accompanied by extensive inflammatory spread (including periappendiceal tissue involvement, abscess formation, or even abdominal cavity diffusion). For low-grade simple appendicitis, antibiotic-based conservative treatment can effectively control local inflammation; in contrast, high-grade complicated cases often respond poorly to simple antibiotic therapy, which easily leads to persistent or recurrent inflammation and ultimately treatment failure, due to the more complex pathological changes of the appendix.

This study further identified that elevated levels of inflammatory indicators, including NLR, PCT, CRP, white blood cell count, and neutrophil count, as well as higher CT grades, are associated with an increased risk of conservative treatment failure. These findings are supported by relevant research: Şahin Kahramanca et al.^17^ demonstrated that preoperative NLR is a valuable parameter for diagnosing acute appendicitis and differentiating simple from complicated cases, and Takayuki Shimizu et al.^18^ pointed out that patients with low NLR are more suitable for antibiotic therapy. The potential mechanism may lie in the fact that increased CT grades and inflammatory index levels are accompanied by severe appendiceal pathological changes, such as obvious swelling, suppuration, gangrene, or perforation. These pathological changes can severely damage the structure and function of the appendix, making it difficult for conservative treatment to reverse the lesions and restore the normal state of the appendix, thus resulting in antibiotic therapy failure.

Appendicitis relapse is another critical factor affecting the clinical decision -making of acute appendicitis treatment. Existing studies have reported that the relapse rate after successful conservative treatment ranges from 5.4 % to 39.5 %,^3^^,^^4^^,^^16^^,^^19^, ^20^, ^21^, ^22^ but relevant research on the risk factors for relapse remains scarce. The present study found that CT grade is an important prognostic factor for relapse after conservative treatment of acute appendicitis: patients with CT Grade 1 had significantly longer relapse-free survival time than those with higher grades, and the risk of relapse increased with the elevation of CT grades. The possible reason is that higher CT grades indicate more severe pathological changes of the appendix; even after successful conservative treatment, these pathological changes are difficult to be completely reversed, leaving potential risk factors for relapse and thus increasing the likelihood of recurrence compared with low-grade cases. In addition, many clinical studies^23^ classify fecalith-related appendicitis as complicated appendicitis and do not recommend conservative treatment, since these patients usually suffer from severe abdominal pain and have a low success rate of conservative treatment. Even if conservative treatment succeeds in individual cases, retained or displaced fecaliths in the appendiceal lumen can still act as an incentive for subsequent relapse.

Guided by CT grading and combined with inflammatory markers, clinicians can more accurately select treatment plans. For patients with low CT grades, conservative treatment can be considered after fully informing the condition and the possibility of relapse, with close observation of the condition. For patients with high CT grades, surgical treatment should be prioritized to avoid delaying the disease due to conservative treatment, leading to further deterioration of appendiceal inflammation, severe complications such as gangrene, perforation, diffuse peritonitis, and even life-threatening situations.

Accurate diagnosis of CT Grade 1/2 simple acute appendicitis is the key prerequisite for formulating conservative treatment regimens. However, false-positive diagnoses may give rise to unfavorable clinical outcomes, thus underscoring the imperative to establish a false-positive discrimination system. From the perspective of inflammatory markers, the levels of CRP and PCT in false-positive cases (e.g., functional abdominal pain or mild intestinal irritation) tend to decrease rapidly. In contrast, these two indicators in patients with true-positive CT Grade 1/2 appendicitis generally remain slightly elevated or decline slowly, which is consistent with the conclusion reported by Gurleyik et al.^14^ Combined with the results of this study, dynamic monitoring of the levels of inflammatory markers (CRP and PCT) during conservative treatment can not only predict the severity of the disease but also serve as an important basis for distinguishing false positives.

From the perspective of physical examinations, abdominal pain in false-positive cases is mostly irregular. Tenderness at McBurney's point alleviates over time without the progression of rebound tenderness. In contrast, tenderness persists in patients with true-positive CT Grade 1/2 appendicitis, and some may present with mild muscle tension (an early sign of peritoneal irritation). Given the physical examination differences between false-positive cases and true-positive patients with CT Grade 1/2 appendicitis, in clinical practice, the exclusion of false positives should be achieved by combining serial monitoring of inflammatory markers with repeated physical examinations.

For CT Grade 1/2 patients who switch to surgical treatment due to failed conservative treatment, postoperative pathological examination is the “gold standard” for ruling out false positives. All 217 patients with treatment failure in this study underwent surgery, and the false-positive rate can be estimated through post-hoc analysis comparing “preoperative CT Grade 1/2 and postoperative pathological results”: this is also the direction of further research.

The exclusion of false-positive CT Grade 1/2 appendicitis relies on a multi -dimensional system of “dynamic indicators + detailed imaging + pathological verification”. Future studies can optimize the design based on this framework to improve diagnostic accuracy.

This study has certain limitations. First, it is a single-center study with a relatively limited sample size, especially the small number of patients in CT Grades 4 and 5, which may affect the universality and accuracy of the research results. In the future, multicenter, large-sample studies are needed to further verify the value of CT imaging grading combined with inflammatory markers in predicting the prognosis of conservative treatment for acute appendicitis. Second, this study only analyzed the relationship between partial clinical indicators and the prognosis of conservative treatment, without considering other factors that may affect the prognosis, such as the patient's underlying diseases, lifestyle, symptoms, etc. In follow-up studies, more relevant factors can be included for comprehensive analysis to establish a more perfect prognostic prediction model. Finally, the diagnosis of Grade 1/2 simple acute appendicitis in this study relies on “spiral CT at admission + clinical symptoms + dynamic detection of inflammatory markers”, which is consistent with the conventional clinical diagnostic criteria for acute appendicitis.^1^^,^^2^ However, a verification system targeting false positives has not been established. The aforementioned limitation may introduce bias into the correlation analysis between “CT grading and the prognosis of conservative treatment”. For instance, incorporating the “low recurrence rate” of false-positive cases into the prognostic data of CT Grade 1/2 may lead to an underestimation of the actual recurrence risk in patients with true-positive Grade 1/2 appendicitis.

In future multicenter studies, it is necessary to integrate “dynamic monitoring + pathological verification + detailed imaging evaluation” to construct a false-positive exclusion system for Grade 1/2 appendicitis. This will enhance the accuracy of research conclusions and their clinical translation value.

## Conclusion

In conclusion, CT imaging grading combined with inflammatory markers (PCT, CRP, etc.) is of great value in predicting the prognosis of conservative treatment for acute appendicitis. Elevated PCT and CRP levels are associated with higher CT imaging grades. CT grade is an important predictor of the prognosis and relapse of antibiotic treatment for acute appendicitis. Patients with high CT grades have a low success rate of conservative treatment and a high risk of relapse. Clinicians should comprehensively evaluate the condition based on CT imaging grading and inflammatory markers to develop individualized treatment plans for patients, so as to improve the treatment effect and patient prognosis.

## Authors’ contributions

The author independently completed all work of this study, specifically including formulating the research concept; conducting data collection and processing raw data; performing data analysis and demonstrating results; drafting the initial manuscript, revising and refining it, and finalizing the manuscript; and assuming full responsibility for the authenticity, scientific validity, and content of the study.

## Funding

This research did not receive any specific grant from funding agencies in the public, commercial, or not-for-profit sectors.

## Data availability

The datasets generated and/or analyzed during the current study are available from the corresponding author upon reasonable request.

## Conflicts of interest

The authors declare no conflicts of interest.
